# Mapping and managing geographic variation in elective surgeries through user-friendly data presentation: insights from Tuscany region

**DOI:** 10.1007/s43999-025-00074-0

**Published:** 2025-09-17

**Authors:** Alessia Caputo, Milena Vainieri, Sabina Nuti

**Affiliations:** 1 Management and Healthcare Laboratory, Institute of Management, Scuola Superiore Sant’Anna, Pisa, Italy; 2 Interdisciplinary Research Center “Health Science”, Scuola Superiore Sant’Anna, Pisa, Italy

**Keywords:** Unwarranted variation, Elective surgery, Systematic component of variation, Performance evaluation system, Healthcare decision-making, Data visualization

## Abstract

Geographic variation in elective surgical procedures poses challenges to healthcare equity, efficiency, and resource allocation. This study investigates variation in 14 elective surgical procedures performed in Tuscany, Italy, in 2022, focusing on both regional and Local Health Authority levels. Using hospital discharge data, we calculated treatment rates and the Systematic Component of Variation (SCV) to quantify unwarranted variation, applying McPherson et al. (1996)‘s thresholds for interpretation. Results revealed substantial differences across procedures, with low SCVs for hip replacement and inguinal hernia repair, and very high SCVs for vein stripping and coronary artery bypass grafting. To support interpretation and governance, we developed a graphical tool that visually represents SCV levels using an intuitive, color-coded format. The tool was designed to reduce the uncertainty healthcare professionals often face when interpreting variation without clear clinical benchmarks, helping them distinguish between acceptable and potentially unwarranted differences. It was presented during the 2023 Tuscan Performance Evaluation System event and pilot-tested in 2025 with 23 healthcare professionals. The tool improved participants’ ability to identify the most appropriate level of governance for action and increased confidence in understanding variation patterns. This study offers a replicable model for analyzing variation and demonstrates the value of user-friendly data visualizations in supporting informed, equity-oriented healthcare decisions. Further validation is recommended to assess long-term impact.

**Supplementary Information:**

The online version contains supplementary material available at 10.1007/s43999-025-00074-0.

## Introduction

Geographic variation in elective surgery has lasting and widespread impacts globally [1, 2], affecting large entities like countries and regions, as well as smaller areas such as hospital service [3–6]. Since the early 20th century, researchers, healthcare managers, and policymakers have worked to understand and address this phenomenon, developing strategies aimed at reducing unwarranted variation. A widely adopted approach has been the creation of healthcare Atlases, which document and analyze differences in medical practice, particularly in elective surgical procedures [3, 7, 8]. These Atlases vary in scope and scale, from one-time publications focusing on a narrow set of indicators to comprehensive, regularly updated systems integrated with clinical, research, and policy frameworks. Insights from these tools reveal that healthcare utilization rates in a given area often remain stable over time, while neighboring regions may differ significantly. This phenomenon, known as the “surgical signature” [9], reflects how local clinical practices and organizational habits affect care delivery. It also shows just how persistent geographic variation can be. However, even when such variation is well documented, it does not always lead to change. This reflects what has been described as an “adoption–use gap”: while data are often adopted and discussed, they are not consistently used to inform decisions or drive action. One key reason for this is the lack of clear standards or actionable targets that would help health professionals and managers understand when variation is acceptable and when it signals underuse or overuse. This gap is further exacerbated by difficulties in interpreting data and linking it to performance goals, limiting the effective use of evidence in the public sector [10]. Without clear communication strategies, commitment to shared objectives may weaken [11, 12]. To address geographic variation in elective surgery effectively, it is essential to develop tools that not only provide detailed analysis but also define clear, actionable goals. Importantly, such targets can only be set for clinically proven and setting-sensitive services [13], as identified in Nuti and Seghieri [14] revision of Wennberg’s classification [15]. For these categories, formal mechanisms such as performance evaluation systems (PESs) can be instrumental in clarifying the goals that healthcare organizations and professionals must pursue. In contrast, evaluating performance in preference-sensitive and supply-sensitive services is more complex, as these categories often lack established standards. As Ouchi (1980) explains, formal mechanisms tend to fail when ambiguity in performance evaluation is high [16]. In such cases, informal mechanisms like “clan control,” which relies on creating goal congruence through socialization and peer review, become essential [16]. Preference-sensitive care, in particular, depends significantly on medical practice, with variation often referred to as medical practice variation [17]. In these cases, physician peer groups can help manage variation through shared learning and collaboration. Socialization and learning, key levers of control [18], are particularly valuable in navigating uncertainty and ambiguity [19]. Whether using formal or informal mechanisms, it is not enough to present absolute figures. A meaningful evaluation of variation is essential. When evidence-based standards are lacking, as with many elective surgeries, assessing geographic variation itself can guide action. However, not all variation is problematic. Efforts should focus on minimizing unwarranted variation, those driven by clinicians’ beliefs or resource allocation, rather than appropriate differences based on patient needs [20]. To distinguish between random variation and systematic variation that can be considered unwarranted, the Systematic Component of Variation (SCV) is a useful measure. SCV values provide a clear framework for evaluation: values exceeding 10 indicate very high variation, those between 5.4 and 10.0 reflect high variation, values between > 3 and < 5.4 suggest moderate variation likely attributable to differences in medical practice styles or discretion, and values below 3 represent low variation [21]. Crucially, how this information is presented matters. Engaging, accessible formats encourage healthcare professionals and managers to move beyond routine interpretations of data. While targets and standards are often necessary for driving decision-making, they may not always be sufficient, particularly in the healthcare sector. This is due to the sector’s inherent complexity, which involves a wide range of stakeholders, intricate relationships, and a delicate equilibrium between formal control mechanisms and the autonomy of healthcare professionals [22–24]. This complexity makes information handling susceptible to biases and misinterpretations, which can hinder effective decision-making. To address this, research on performance representation emphasizes the importance of simplifying data through visual tools, benchmarks, and intuitive reporting systems. These approaches improve understanding, retention, and the likelihood of taking action [25–27]. Since performance evaluation systems are designed to support organizational strategy, how data is represented affects the use of performance metrics and the achievement of the end-results these systems are designed to oversee [28, 29].

Building on these premises, the aim of this study is to understand how variation in elective surgery, measured through the SCV, can be used to generate insights in areas where clinical guidelines or benchmarks are lacking. We focus on procedures that are relevant for health system governance but do not have clear standards for what constitutes an appropriate rate of treatment. In this context, SCV offers a way to identify patterns of unwarranted variation. Using hospital discharge records from 2022 in the Tuscany region, the goal is to translate this analysis into a practical and accessible graphical tool that can help policymakers and healthcare professionals identify where targeted interventions may be needed. By doing so, the study seeks to facilitate the identification of strategies that various levels of governance—policymakers, healthcare managers, and providers—should implement to address these issues. Drawing on the importance of PESs and effective communication channels for sharing information and fostering commitment across organizational levels, the study introduces an innovative data representation to highlight and address potential unwarranted variation. In brief, this study is aimed at regional and local health managers, policymakers, and clinical directors who rely on the Tuscan PES to inform both strategic planning and day-to-day operational decisions. Although data on treatment variation are regularly shared within the system, there is often a gap between having access to this information and being able to translate it into clear, actionable decisions, especially in cases where no formal benchmarks or clinical standards exist. The graphical tool developed in this study is intended to help bridge that gap by offering a simple and intuitive way to interpret variation, making it easier to identify where targeted interventions may be needed. This paper is organized as follows. The next section outlines the methods, detailing the study setting and the adopted methodology. The third section investigates the results of the analysis. Discussions and conclusions are then developed in the final sections.

## Methods

### Study setting

**Fig. 1:**
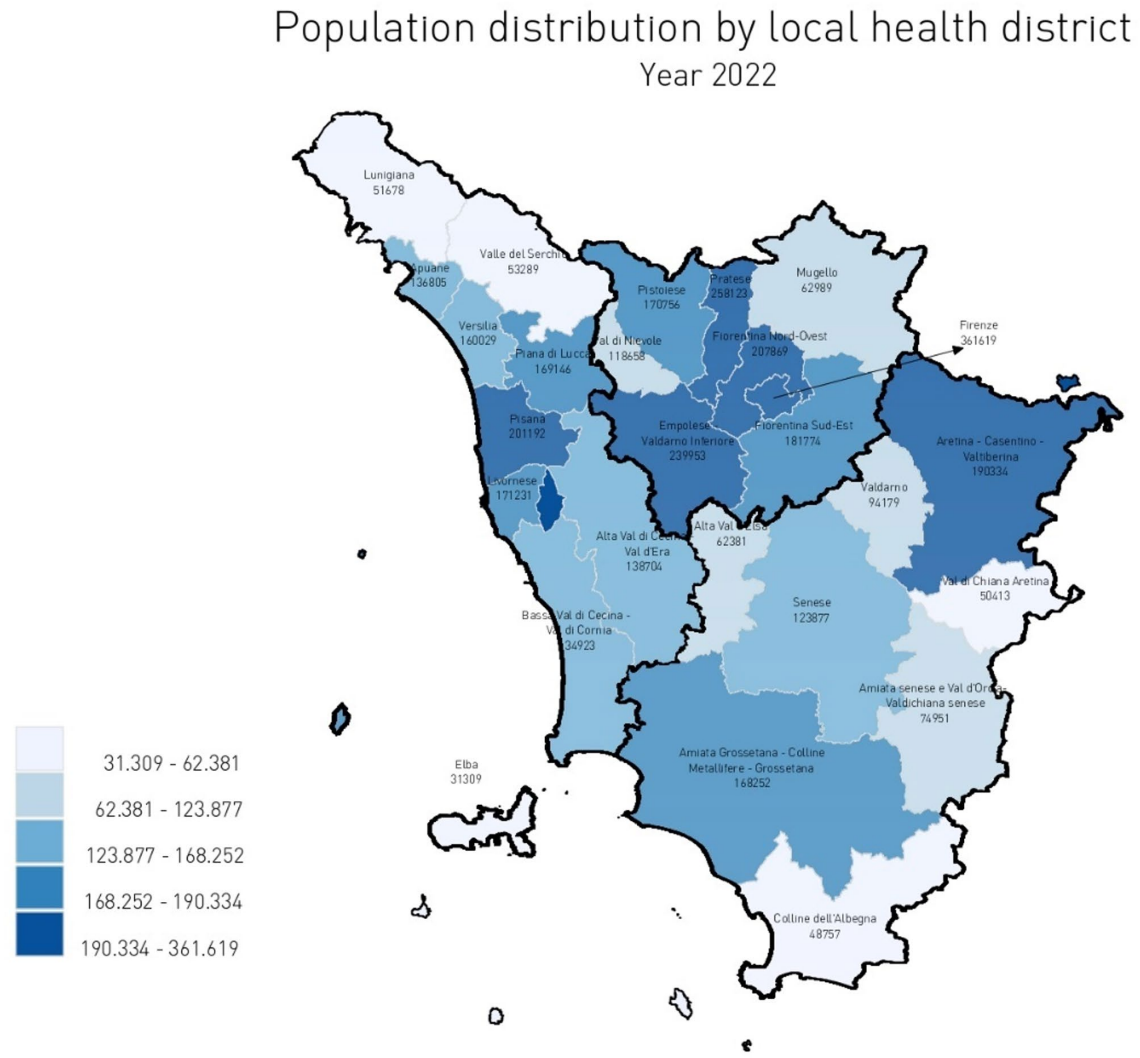
Population distribution by Tuscan LHDs in year 2022

This study builds upon the 20-year experience of the Tuscan PES, a regional tool designed to assess healthcare performance in the Tuscany region. As such, it can be regarded as a longitudinal case study. The Italian National Health Service (*Servizio Sanitario Nazionale* [SSN]), a decentralized Beveridge-type model, delegates healthcare organization and delivery to the regions [30, 31]. Consequently, regional and interregional performance evaluation systems, including the Tuscan PES, have been developed to address these responsibilities. Located in central Italy, Tuscany serves a population of 3.7 million residents and accounts for approximately 6% of the national healthcare fund, equating to around 7 billion euros in 2021. The healthcare system is structured into three Local Health Authorities (LHAs)—North-West, Center, and South-East—and five independent health authorities: three Teaching Hospitals (THs) in Pisa, Siena, and Florence, and two Research Hospitals. While the THs and Research Hospitals operate independently without territorial services, the LHAs are responsible for public health, community services, and primary care, encompassing both hospital and territorial facilities organized into 26 Local Health Districts (LHDs). The LHAs’ finances are managed through a capitation-weighted budgeting system, with certain LHDs, particularly those around Florence, serving densely populated areas exceeding 360,000 residents (Fig. 1).

In 2004, the Tuscany Region launched its PES to foster dialogue among healthcare stakeholders [32]. This multidimensional tool tracks over 700 key performance indicators (KPIs) across areas such as equity, population health, emergency care, satisfaction, governance, and quality. Indicators are assessed using a five-color band from red (critical) to dark green (excellent), helping quickly identify strengths and weaknesses [12]. The PES is built on principles of evidence-based data, benchmarking, transparency, and timeliness. Benchmarking, particularly important for public service monopolies, promotes mutual learning and collaborative problem-solving rather than punitive actions [13, 33]. Public disclosure further enhances transparency, supports peer review, and bolsters professional reputation [24]. Since 2010, the Tuscan PES has monitored geographic variation in elective surgeries by benchmarking performance across hospitals and LHAs, using ascending histograms and the coefficient of variation (CV) to summarize regional differences, though intra-LHA variation is not statistically analyzed. Each year, a series of meetings is held with management representatives, CEOs, and healthcare professionals from the health authorities to review benchmarking data and identify targeted actions to address unwarranted variation. While the PES has influenced behavior through goal-setting and discussion, unwarranted variation remains in some elective procedures due to the lack of clear clinical standards. In these cases, although variation was shown, it was not evaluated using the red-to-green scale, limiting the system’s capacity to guide targeted interventions. One of the recurring challenges has been understanding at which level governance decisions should be made, especially considering the hierarchical structure of the Tuscan healthcare system. Local health districts are grouped into local health authorities, which in turn report to a regional central authority. This structure raises questions about whether certain issues require a response at the regional level, such as a policy change or the reallocation of resources, or whether they are better addressed locally within individual health authorities.

### Study design

The study followed a stepwise analytical procedure aimed at identifying and analyzing unwarranted geographic variation in elective surgical procedures. We first selected relevant procedures, then extracted and analyzed hospitalization data, and finally developed and tested a visual tool to support governance decisions. We conducted a descriptive cross-sectional study to measure geographic variation in elective surgical procedures using health administrative data from Hospital Discharge Records in the Tuscany region. Hospitals routinely submit inpatient data to the Regional Health Information System Office, which ensures data quality and anonymizes patient records using unique encrypted identifiers. These anonymized data were shared with our research laboratory under a collaboration agreement with the Tuscany Region Health Authority (regional resolution 159/2019). The study complies with European GDPR (2016/679) and Italian Privacy Law (101/2018), with authorization from the Italian Data Protection Authority for the use of anonymized administrative data for research since 2012. To enhance reporting quality, transparency, and replicability, we applied the REporting of studies Conducted using Observational Routinely collected Data (RECORD) checklist [34]. Population information is sourced from the Italian National Institute of Statistics (ISTAT).

The analysis involved all patients with planned hospitalizations for specific surgical procedures between January 1 and December 31, 2022. In line with Stewart et al. (2024) [35], who note that elective procedures are generally considered to be nonessential or nonemergent, we defined elective surgery as planned, non-urgent hospitalizations, whether or not preceded by pre-hospitalization. This definition was operationalized using administrative admission codes, which allowed us to systematically include only non-urgent cases and exclude emergency procedures. Additionally, supplementary analyses were conducted for the years 2019, 2020, and 2021 to identify potential differences in trends across the pre-COVID-19, during-COVID-19, and post-COVID-19 periods (see Supplementary Material). The paper examined geographic variation in fourteen procedures that typically exhibit significant unwarranted differences both across and within countries or regions. These fourteen procedures were selected because they are clinically and organizationally relevant yet lack well-established thresholds or evidence-based standards for what constitutes an optimal treatment rate. This absence of formal guidelines makes them particularly suitable for variation analysis using the SCV, which can help identify potential underuse or overuse instances. In addition, all 14 procedures are routinely monitored by the Tuscan PES, and are already well-known to regional policymakers and healthcare professionals, making them practically meaningful for informing governance and performance improvement initiatives. These procedures included carotid endarterectomy (CEA), cholecystectomy, colectomy, coronary angiography, coronary angioplasty, CABG, hemorrhoidectomy, hip replacement, hysterectomy, inguinal hernia repair, knee replacement, laparoscopic cholecystectomy, tonsillectomy, and vein stripping (details about ICD-9-CM (International Classification of Diseases, 9th revision - Clinical Modification) are reported in the Supplementary Material Table S1). Data processing was performed using SAS version 9.4 (SAS Institute Inc., Cary, NC), with local health districts (LHDs) at the local health district (LHD) level, with regional data matching. No person-level record linkage was performed.

### Hospitalization rates and variation in access to surgical care

Crude and age-and-sex-standardized surgical hospitalization rates were calculated to assess the level of variation across LHDs (*n* = 26). Treatment rates were defined as the number of procedures performed for residents of each LHD per 100,000 population. These rates included hospitalizations of Tuscan residents regardless of the hospital location. Standardization was performed by employing a 5-year age grouping, according to the standard population provided by the Tuscany region and the 2013 Eurostat guidelines. Numerators and denominators included male and female patients over 18 years old, with the exception of hysterectomy (females over 18 only), hip replacement (patients above 65), and tonsillectomy (patients under 18), in line with Tuscan PES KPI definitions [36]. To assess the proportion of variation among health districts that is systematic and extends beyond expected random variation, we computed the Systematic Component of Variation (SCV), following approaches used in comparable studies [37–40]. The SCV involves an indirect standardization method where sex-and-age grouping is assumed to alter the surgery risk by a fixed multiplicative factor [41]. The SCV quantifies the relative systematic component of variation among health districts by deducting the random component of variance (variance within districts) from the total variance estimate [21, 41]. Therefore, the SCV denotes the systematic variation deemed to be beyond chance, hence presumably unwarranted [3]. We calculated the SCV for the entire region, considering all 26 LHDs. We also calculated it individually for each of the three Tuscan LHAs (North-West (LHA1), Center (LHA2), South-East (LHA3)), considering only the LHDs belonging to each specific LHA. Taking *K* as the number of Tuscan LHDs, *O*_*i*_ as the observed number of hospitalizations for each surgical procedure, and *E*_*i*_ as the expected number of hospitalizations for each surgical procedure given the regional or LHA age- and sex-specific rates applied to the age-sex distribution of each district, the SCV was calculated as follows [41]:

$$\:SCV=\frac{1}{K}\left(\sum\:_{i=1}^{k}\frac{({O}_{i}-{E}_{i}{)}^{2}}{{{E}_{i}}^{2}}-\sum\:_{i=1}^{k}\frac{1}{{E}_{i}}\right)*100$$

### Graphical representation of variation and dissemination of results

**Fig. 2:**
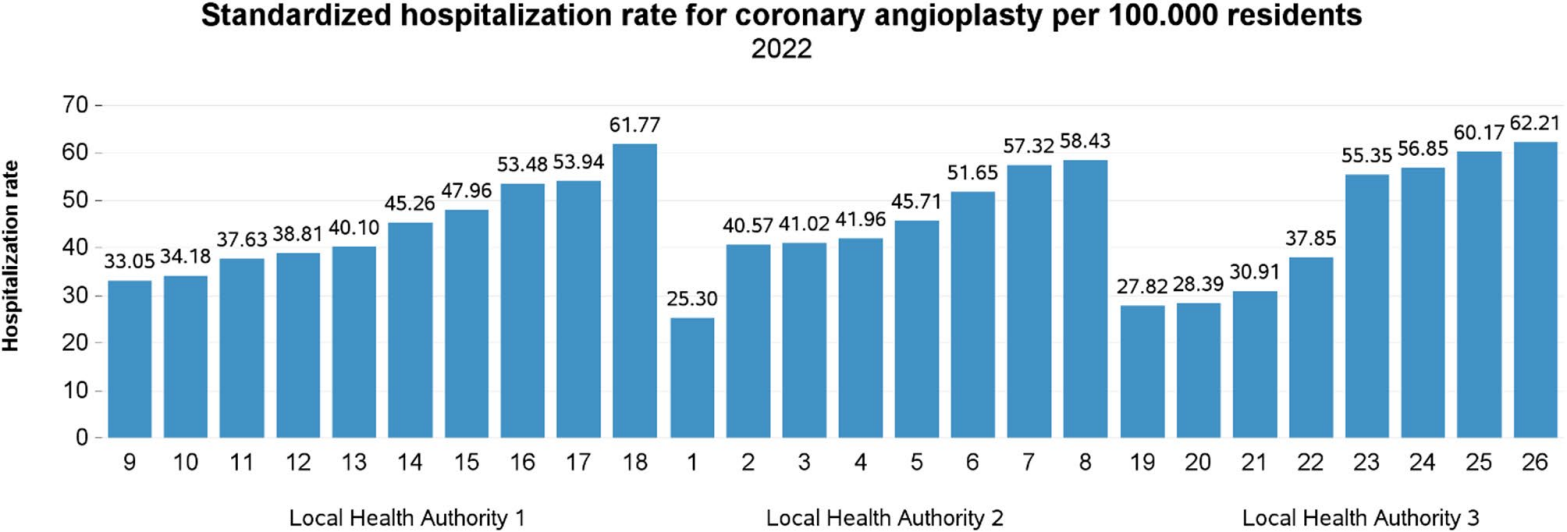
Standardize hospitalization rate for coronary angioplasty per 100.000 residents, 2022

After computing the SCV for the selected surgical procedures, we developed a visual tool to make geographic variation data more accessible to healthcare stakeholders. The tool aims to support policymakers and professionals in identifying unwarranted variation and in setting thresholds for underuse or overuse across 14 elective procedures. First, we established SCV-based benchmarks using the classification by McPherson et al. [21],, categorizing variation as low (< 3), moderate (3–5.4), high (5.4–10), and very high (> 10). Second, based on these thresholds, we created a color-coded graphical representation showing SCV levels at both regional and LHA scales, allowing for quick identification of treatment rates requiring targeted action. The graphical representation was showcased during the Tuscan PES results disclosure on June 1, 2023. This annual event promotes transparent, evidence-based decision-making among policymakers, healthcare managers, and professionals. The event, with its large, engaged audience already accustomed to discussion about mitigating unwarranted variation, was ideal for validating the visualization tool. To understand how well the tool worked in practice, we ran a pilot test in February 2025 with 23 healthcare professionals from across Tuscany. The group included staff from all three LHAs, involved in roles such as clinical leadership, strategic planning, and quality improvement. The goal was to see whether the new visual format helped them more clearly identify the most appropriate level of governance for action, that is, whether a given variation should be addressed at the regional level (through policies or broader coordination) or within a specific local health authority (through internal changes or peer-led initiatives). Participants first reviewed traditional histograms for hysterectomy and coronary angioplasty, grouped by LHA (see Fig. 2 for an example). They were then asked to answer the question: *“At what level of governance should intervention take place to manage the avoidable variation in (i) hysterectomy and (ii) coronary angioplasty?”*. Subsequently, the same participants were presented with the new graphical representation and asked the same question. This two-step approach allowed for a first comparative evaluation of whether the innovative visual tool enhanced stakeholders’ ability to detect unwarranted variation and more accurately determine the appropriate level of governance, namely regional and/or LHA, for intervention.

## Results

Based on the inclusion criteria outlined in the methods section, Table 1 presents the annual regional standardized and crude treatment rates and regional SCVs for various surgical procedures. In 2022, *hip replacement* was the most frequently performed procedure, with a rate of 383.05 per 100,000 population, while *coronary artery bypass surgery* (CABG) was the least frequent, with a rate of 15.55 per 100,000 population. Geographic variation in treatment rates was initially assessed using the minimum-maximum range, which, while informative, is susceptible to bias from extreme values. The widest variations were observed in *hip replacement* (303.08–476.09), *inguinal hernia repair* (206.02–367.17), and *coronary angiography* (79.77–229.53), with differences of 172.01, 161.15, and 149.76, respectively. To address the limitations of range-based metrics and the sensitivity of crude treatment rates to population size, we employed a more robust measure, the SCV, to assess unwarranted variation both across geographic areas and between procedures. This analysis confirmed the ubiquity of geographic variation (Table 1). The procedures with the highest SCV values at the regional level in 2022 were *CABG* (52.34), *vein stripping* (47.11), *hemorrhoidectomy* (12.89), *tonsillectomy* (9.74), *carotid endarterectomy* (9.53), *coronary angiography* (6.30), and *hysterectomy* (6.09). According to McPherson et al. [21] these SCV values fall within the high or very high categories. Results for LHAs are shown in Supplementary Table S2. For clarity and brevity, the focus of this section is limited to regional-level findings. As for temporal trend analysis (Supplementary Table S3), both regional and LHAs variation measured through SCV across remains persistently high across all four years for the selected procedures.

**Table 1:** Crude regional treatment rates (per 100,000 residents) and SCV by procedure in 2022

Procedure	Standardized rate		Crude rate (*n*)	Min – Max crude rate	SCV
Carotid endarterectomy	24.16		28.94 (905)	9.87–53.91	9.53*
Cholecystectomy	171.73		179.98 (5,629)	132.00–239.55	2.36
Colectomy	31.22		37.28 (1,166)	26.20–48.14	4.81
Coronary angiography	127.53		150.44 (4,705)	79.77–229.53	6.30*
Coronary angioplasty	46.10		53.27 (1,666)	29.57–74.68	3.41
Coronary artery bypass surgery	12.42		15.55 (455)	5.72–36.83	52.34†
Hemorrhoidectomy	47.90		48.03 (1,502)	23.67–95.69	12.89†
Hip replacement	391.44		383.05 (3,511)	303.08–476.09	0.43
Hysterectomy	106.09		109.25 (1,777)	29.10–210.27	6.09*
Inguinal hernia repair	240.13		272.58 (8,525)	206.02–367.17	2.44
Knee replacement	206.47		230.76 (7,217)	166.70–305.31	3.85
Laparoscopic cholecystectomy	161.98		164.32 (5,139)	118.70–228.09	3.06
Tonsillectomy	117.66		98.18 (526)	58.66–204.61	9.74*
Vein stripping	36.38		37.03 (1,158)	18.35–111.58	47.11†

† Very high variation (SCV > 10)

* High variation (SCV between 5.4 and 10)

### Discovering patterns in geographic variation of treatment rates

The geographic variation observed at the regional level highlights significant differences in the utilization of elective surgical procedures LHDs and LHAs. Addressing this issue requires targeted action from healthcare authorities, managers, and CEOs. To bring these disparities to light and stimulate action, we presented our findings during the Tuscan PES result disclosure event on June 1, 2023. The presentation was well-received, garnering positive oral feedback and sparking constructive discussions among participants. Our approach to presenting the data unfolded in two distinct steps. First, we provided an overview of treatment rates by procedure, disaggregated by LHDs and grouped according to the LHAs they belonged to, alongside the regional and LHA-level SCVs (see Fig. 2 for an example). Figure 2 shows the standardized hospitalization rates for coronary angioplasty across all 26 local health districts. The chart follows the standard visual style used in the Tuscan PES, where all bars are displayed in the same color and no evaluation criteria are applied. While this format makes it easy to compare rates across areas, it does not indicate whether the differences are expected, appropriate, or potentially unwarranted, providing no guidance on interpretation. This step aimed to offer a granular understanding of variations both within and across LHAs, enabling participants to identify areas of concern.

**Fig. 3:**
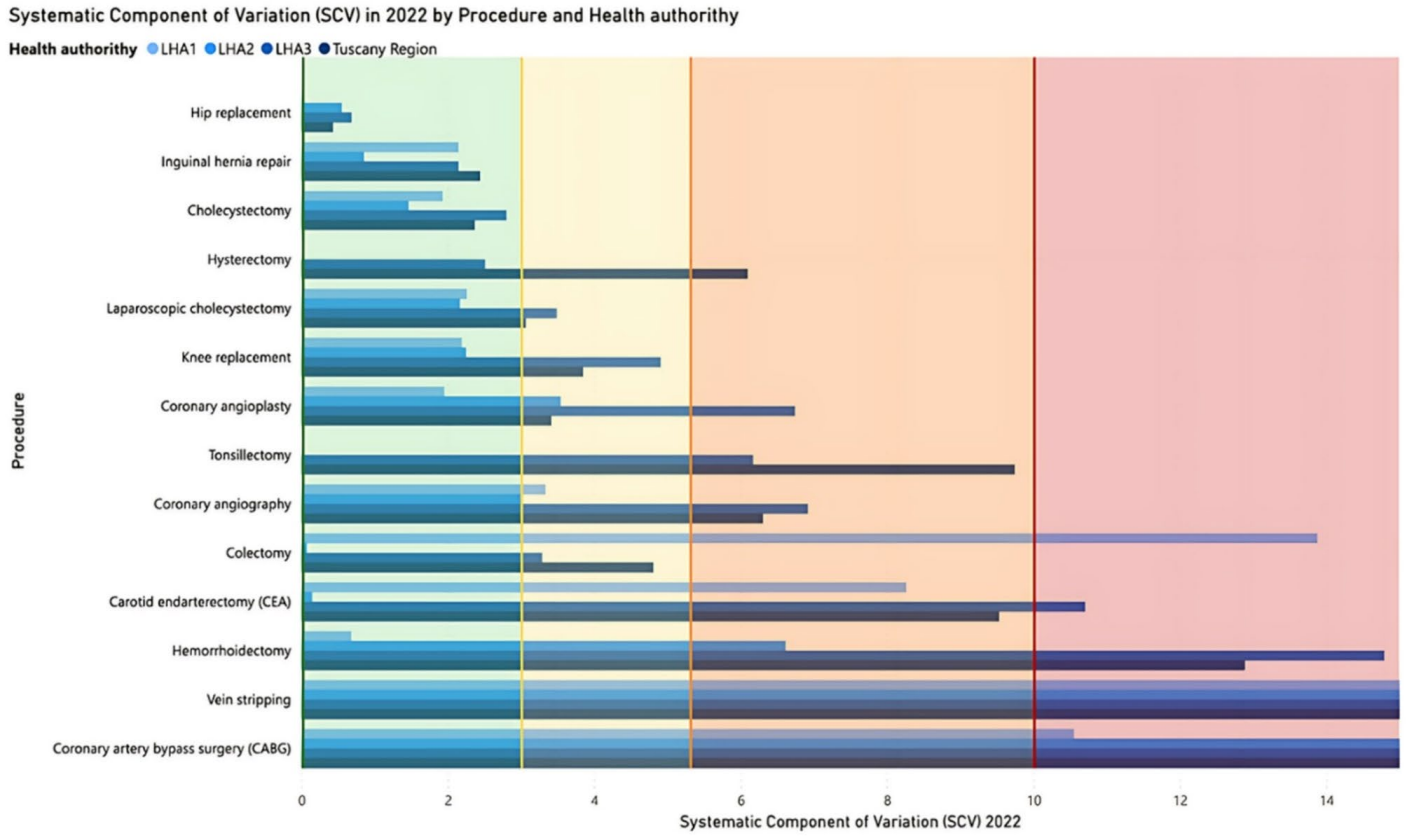
Graphical representation of the 2022 SCV with evaluation colors for fourteen procedures

Next, we introduced an innovative graphical representation designed to serve as a comprehensive snapshot of SCVs by surgical procedure across the region and LHAs (Fig. 3). This visualization employed horizontal bars to represent SCVs for regional and LHA levels, using four shades of blue to differentiate health authorities. To enhance interpretability, the bars were overlaid with colored bands indicating SCV categories, as defined by McPherson et al.: red for very high variation, orange for high variation, yellow for moderate variation, and green for low variation. This design enabled direct and intuitive comparisons across health authorities for each surgical procedure, emphasizing the extent of unwarranted variation. What makes this graph innovative is its ability to turn complex statistical data into a clear and actionable visual format. Rather than simply displaying variation, it links SCV values to defined thresholds of concern, allowing users to immediately see which procedures may require attention and at what level of the system. The graph was developed through an iterative process that began with preliminary SCV results and was refined through internal discussions by the research team. Its design draws inspiration from familiar visual formats used in the Tuscan PES, adopting the same color scheme typically applied to indicators with established clinical guidelines [36].

When comparing regional SCVs with those at the LHA level, three distinct patterns of geographic variation emerged, each providing a deeper understanding of healthcare delivery dynamics. The first pattern, referred to as “Uniformity in Variation”, was characterized by consistently low SCVs across both regional and LHA levels. This pattern, observed in procedures such as hip replacement, inguinal hernia repair, and cholecystectomy, reflected coherent treatment practices across the region, with minimal unwarranted variation. The second pattern, “Localized Drivers of Regional Disparities”, highlighted scenarios where high variation in one or two LHAs disproportionately influenced the regional SCV. For instance, laparoscopic cholecystectomy and knee replacement displayed low variation in two LHAs but moderate variation in a third resulting in a regional SCV classified as moderate. Similarly, coronary angiography’s regional SCV was driven by high variation in LHA3, while hemorrhoidectomy’s very high regional SCV and CEA high regional SCV were attributed to significant geographical variation in LHA3, alongside high variation and low variation in the other two. CABG was a notable case within this pattern, as all three LHAs exhibited very high SCVs; however, one LHA had an exceptionally high SCV that disproportionately influenced the regional SCV. The third pattern, “Inter-LHA variation”, represents the most distinctive scenario and is exemplified by procedures such as hysterectomy, tonsillectomy, and vein stripping. In these cases, the regional SCV exceeds the SCVs observed in any of the three LHAs, highlighting persistent differences in surgical practices between LHAs rather than within them. This suggests that while the LHAs were able to effectively control internal geographic variation by homogenizing treatment rates across health districts within their respective boundaries, they adopted markedly different surgical approaches when compared to one another. The resulting inter-LHA variation was substantial enough to elevate the regional SCV, classifying hysterectomy as high variation, while hemorrhoidectomy and vein stripping fell into the very high variation category.

In the pilot test, the new graphical representation proved more effective in helping healthcare professionals identify the appropriate level of governance for addressing unwarranted variation. For hysterectomy, where the appropriate level of intervention is the regional level, the percentage of correct responses increased from 60.87% with the traditional histogram to 72.73% with the new visual tool. A more substantial improvement was observed for coronary angioplasty, where action is required specifically at the level of LHA 3. In this case, correct answers rose from 4.35 to 40.91%. In both procedures, the share of “I don’t know” responses dropped from 4.35% with the traditional representation to 0% with the new one.

## Discussions

Numerous studies have highlighted the influence of geographic variation on both patients’ well-being and healthcare expenditures [20, 42–45]. Analyzing such variation is essential for identifying opportunities to improve performance, reduce costs, and ensure equitable access to care [46]. Nevertheless, not all variation is detrimental. For this reason, the information shared with healthcare stakeholders should aim to highlight the unwarranted component of variation as accurately as possible. There is a need to report rates using statistical measures capable of estimating the true, non-random component of variation. In this study, we investigated the geographic variation in 14 elective surgical procedures using the SCV to quantify systematic variation. Significant variation was found in most procedures, both regionally and within LHAs. Exceptions included hip replacement, inguinal hernia repair, and cholecystectomy, which showed minimal unwarranted variation. Other procedures, such as CABG and vein stripping, displayed high to very high SCVs in at least one LHA. These results align with broader trends of declining rates in some elective procedures due to evolving clinical guidelines and innovation in less invasive techniques, and pandemic-related restrictions [47–49]. The COVID-19 pandemic further contributed to these reductions, as restrictions on elective surgeries impacted procedural volumes worldwide [50–52]. Despite these declines, our SCV analysis reveals persistent territorial variation in the Tuscany region. Due to the difficulty in identifying thresholds for underuse and overuse in elective surgery rates, evaluating unwarranted variation can help ensure equitable access to care and performance improvements. The study underscores the importance of establishing clear targets to guide stakeholders toward desired objectives in addressing unwarranted variation. While the Tuscan PES has a rich history of informing stakeholders about geographical variations in treatment rates, the absence of a tailored evaluation on the topic risks limiting discussions to “a mere intellectual curiosity” [53]. Without consensus on targets and goals, action may not follow. Since 2010, variation was presented through histograms ordered by increasing rates, without highlighting what portion of it required action. These visuals often sparked curiosity but rarely drove change. To address this, we developed a new graphical representation based on SCV thresholds to classify variation as low, moderate, high, or very high [21]. This approach aimed to reduce cognitive load while enhancing the interpretability and usability of data for healthcare decision-makers [25]. The tool engaged stakeholders more effectively than traditional visuals, helping to raise awareness as a first step toward change. The three variation patterns identified in our analysis help differentiate between systemic issues and differences in local clinical approaches. This distinction is crucial for tailoring interventions: regional issues may require policy changes, while local variation driven by differing clinical approaches might call for targeted educational efforts or harmonization of treatment protocols. The pilot test confirmed the usefulness of the new representation in supporting decision-making. Compared to traditional histograms, it enabled professionals to more accurately determine the right level of governance for intervention, that is, whether action was needed at the regional or LHA level based on the SCV analysis. Although we did not directly measure engagement or perceived cognitive load, the increase in correct responses and the complete disappearance of “I don’t know” answers suggest that the tool made it easier to interpret the data and connect it to governance decisions. These findings support our claim that the tool helps users identify the right level of intervention and makes complex variation patterns more understandable and actionable for healthcare decision-makers. This was particularly clear in the case of coronary angioplasty, where ascending-order histograms failed to distinguish the unwarranted portion of variation. The SCV-based representation filled this gap by offering clear visual cues. In the case of hysterectomy, where variation followed an “Inter-LHA” pattern, the differences between LHAs were already somewhat evident using grouped histograms. However, the new tool made the nature and extent of the variation more immediately understandable. It also reduced uncertainty, with “I don’t know” responses dropping to zero and improved the accuracy of responses overall. In essence, the new graphical representation translates complex data into a more accessible and actionable format. It supports a shift from passive observation to informed, targeted intervention at the appropriate level of governance. While the pilot test was promising, further validation is needed. Future studies should assess users’ perceived understanding and actual retention of the information, ideally through a randomized controlled trials, Discrete Choice Experiments or Experimental Vignette studies [25, 54, 55]. Additionally, prospective studies could assess whether the tool influences clinical practice.

Although this study focuses on the Tuscany region, the methodological approach developed is transferable to other healthcare systems. Geographic variation in elective surgical procedures is a well-recognized phenomenon across a range of countries, including Spain, Canada, the United Kingdom, Germany, and the United States [2]. The challenge of identifying which variations are unwarranted is common to systems where service delivery and resource allocation are decentralized or managed at the regional level. What distinguishes our contribution is the specific use of the Systematic Component of Variation (SCV) not only as a tool for measurement, but also as a means to support governance and target setting. While SCV has been used in previous studies, we applied it to assess whether observed variation may reflect potential underuse or overuse, and to clarify where intervention might be most appropriate. This application can help define performance benchmarks and inform clinical or policy guidelines, particularly in areas where international or national standards are lacking. Because the method relies on routinely collected administrative data, it can be adapted to other settings with similar infrastructures.

To our knowledge, this study is the first that compares the utility of the visual representations of geographic variation clearly providing an assessment. The evaluative thresholds, still based on the 1996 classification, remain the most widely accepted reference for interpreting variation. This is especially valuable where no established standards exist for hospitalization rates. By supporting more informed decision making at the LHA level, where resource allocation has the greatest impact, the study introduces a new graphical tool to identify areas needing targeted governance and intervention. In addition to its methodological contribution, the visual representation introduced in this study addresses a common challenge in health system governance: how to make complex performance data more interpretable and actionable. Many health systems struggle not only with understanding the data but also with determining who should respond to it [56]. By clearly illustrating both the magnitude of variation and the level at which it occurs, the tool helps clarify whether action should be taken locally, regionally, or system-wide. While especially relevant in decentralized systems such as those in Spain, Canada, and the United Kingdom, the approach also holds value in more centralized systems, where clearer communication of performance data can strengthen accountability and decision-making. In this sense, the study contributes to a broader international effort to move beyond monitoring variation toward actively managing it through informed, targeted action.

There are some limitations to acknowledge when interpreting these findings. First, the study does not explore the causes of geographic variation, as its goal was to describe rates and variation, not their underlying drivers. Addressing this would require a different study design and additional patient- and system-level data. Nonetheless, we acknowledge the importance of investigating potential drivers such as supply capacities, clinical practice patterns, and socioeconomic factors. Future research should build on these findings to explore the underlying causes of the observed variation. Second, the analysis relies on administrative data, which, while commonly used in research [38, 44, 45, 57], can include biases from data entry or reporting inconsistencies. However, Tuscany’s health data are routinely validated, reducing this risk. Third, the new graphical representation has not yet undergone formal validation and should be further tested. Lastly, while alternative metrics like the Empirical Bayes (EB) variance component could have been used, we selected SCV for its interpretability. EB, though statistically robust, can be harder for non-specialists to understand and is better suited for small datasets, which was not the case in this study.

In conclusion, this study shows significant and persistent geographic variation in the use of 14 elective surgical procedures in Tuscany, corroborating the concept of “surgical signatures.” Using the SCV helped identify the non-random part of this variation and introduced a way to evaluate and manage unwarranted differences. While further validation is needed, the new user-friendly graphical tool is designed to reduce cognitive effort for healthcare stakeholders and support more informed decision-making.

## Supplementary Information

Below is the link to the electronic supplementary material.

Supplementary Material 1
