# Supplementary material for: Mapping and managing geographic variation in elective surgeries through user-friendly data presentation: insights from Tuscany region
Source: Res Health Serv Reg. 2025 Sep 17;4:14. doi: 10.1007/s43999-025-00074-0 (PMC12440846; doi:10.1007/s43999-025-00074-0)
Supplement: Supplementary file 1 — Supplementary Material 1 [file 43999_2025_74_MOESM1_ESM.docx]

**Appendix**

**Table A1** ICD-9-CM by procedures

| **Procedure** | **ICD-9-CM Code(s)** |
| --- | --- |
| Carotid Endarterectomy (CEA) | 38.12 |
| Cholecystectomy | 51.21 - 51.24 |
| Colectomy | 45.71 – 45.76, 45.79, 45.8, any diagnosis 153* |
| Coronary Angiography | 88.55 - 88.57 |
| Coronary Angioplasty | 00.66, 36.06, 36.07 |
| Coronary Artery Bypass Graft (CABG) | 36.10–36.17, 36.19 |
| Hemorrhoidectomy | 49.46, 49.49 |
| Hip Replacement | 00.70 - 00.73, 00.85 - 00.87, 81.51 - 81.53 |
| Hysterectomy | 68.3*–68.9 Excluded primary diagnosis with the following codes: 179, 180.*, 181, 182.*, 183.*, 184.*, 869.*, 2331, 2332, 2333, 2360, 2361, 2362, 2363, 8674, 8675, 8676, 8677, 8678, 8679, 86800, 86803, 86804, 86809,86810, 86813, 86814, 86819, 8796, 8797, 8798, 8799, 9060, 9081, 9082, 9391, 9474, 23330, 23331, 23332, 23339 |
| Inguinal Hernia Repair | 53.00–53.05, 53.10 - 53.17 |
| Knee Replacement | 81.54 Excluded primary or secondary diagnosis with the following codes: from 800* to 959* |
| Laparoscopic Cholecystectomy | 51.23, any diagnosis 574x and 575x |
| Tonsillectomy | 28.2, 28.3 |
| Vein Stripping | 38.5 |

Based on the inclusion criteria outlined in the methods section of the main text, we displayed in Table A2 the annual crude treatment rates by surgical procedure. For the sake of summary, we will discuss only 2022 results. Findings from 2019 to 2021 should be interpreted accordingly. In 2022, the crude utilization rate for carotid endarterectomy ranged between 9.87 and 53.91 in the 26 LHDs (regional treatment rate: 28.94/100,000 inhabitants, n=905); for cholecystectomy between 132.00 and 239.55 (regional treatment rate: 179.98/100,000, n=5,629 patients); for colectomy between 26.20 and 48.14 (regional treatment rate: 37.28/100,000, n=1,166); for coronary angiography between 79.77 and 229.53 (regional treatment rate: 150.44/100,000, n=4,705); for coronary angioplasty between 29.57 and 74.68 (regional treatment rate: 53.27/100,000, n=1,666). Minimum and maximum treatment rates in the 26 Tuscan LHDs were 5.72 - 36.83 for CABG y (regional treatment rate: 15.55/100,000, n=455); 23.67 - 95.69 for hemorrhoidectomy (regional treatment rate: 48.03/100,000, n=1,502); 303.08 - 476.09 for hip replacement (regional treatment rate: 367.56/100,000, n=3,511); 29.10 - 210.27 for hysterectomy (regional treatment rate: 109.25/100,000, n=1,777). Surgical treatment rates for inguinal hernia repair varied between 206.02 and 367.17 (regional treatment rate: 272.58/100,000, n=8,525); for knee replacement, between 166.7 and 305.31 (regional treatment rate: 230.76/100,000, n=7,217); for laparoscopic cholecystectomy between 118.7 and 228.09 (regional treatment rate: 164.32/100,000, n=5,139); for tonsillectomy between 58.66 and 204.61 (regional treatment rate: 98.18/100,000, n=526); for vein stripping between 18.35 and 111.58 (regional treatment rate: 37.03/100,000, n=1,158).

Computing the range, namely the difference between the highest and lowest values, provides an initial overview of geographic variation in treatment rates, albeit prone to bias from extreme values. Examining the boxplots in Figure A1, we observed a persistent geographic variation over time. The boxplots consistently displayed similar dimensions across the years, indicating comparable distributions of the lower quartile, median, and upper quartile. Furthermore, the length of the whiskers remained steady over the four years, suggesting that outlier LHDs tended to maintain historical treatment rates rather than aligning with neighboring LHDs' rates. This observation supports the "surgical signatures" principle, where idiosyncrasies persist at the LHD level. The notable exception was inguinal hernia repair, which exhibited less variability among LHDs post-COVID-19 — a trend that has consolidated in 2022.

**Table A2** Annual crude regional treatment rates (per 100,000 residents) by procedure

| **Procedure** | **2019** | | **2020** | | | **2021** | | | **2022** | | |
| --- | --- | --- | --- | --- | --- | --- | --- | --- | --- | --- | --- |
|  | **Rate (n)** | **Min - Max** |  | **Rate (n)** | **Min - Max** |  | **Rate (n)** | **Min - Max** |  | **Rate (n)** | **Min - Max** |
| Carotid endarterectomy | 33.07 (1,048) | 14.00 - 55.34 |  | 24.55 (772) | 7.54 - 51.07 |  | 27.47 (865) | 11.24 - 60.33 |  | 28.94 (905) | 9.87 - 53.91 |
| Cholecystectomy | 188.13 (5,962) | 131.41 - 321.12 |  | 119.40 (3,755) | 75.42 - 166.19 |  | 140.01 (4,409) | 105.44 - 237.36 |  | 179.98 (5,629) | 132.00 - 239.55 |
| Colectomy | 41.87 (1,327) | 24.66 - 59.23 |  | 34.44 (1,083) | 23.78 - 51.17 |  | 39.41 (1,241) | 23.50 - 51.71 |  | 37.28 (1,166) | 26.20 - 48.14 |
| Coronary angiography | 169.35 (5,367) | 81.59 - 289.18 |  | 132.41 (4,164) | 52.53 - 206.19 |  | 153.67 (4,839) | 55.61 - 238.04 |  | 150.44 (4,705) | 79.77 - 229.53 |
| Coronary angioplasty | 64.25 (2,036) | 28.11 - 100.23 |  | 47.28 (1,487) | 20.47 - 82.72 |  | 55.57 (1,750) | 18.84 - 83.43 |  | 53.27 (1,666) | 29.57 - 74.68 |
| Coronary artery bypass surgery | 15.24 (483) | 6.84 – 35.00 |  | 13.16 (414) | 3.74 - 47.42 |  | 14.64 (461) | 3.15 - 33.30 |  | 15.55 (455) | 5.72 - 36.83 |
| Hemorrhoidectomy | 52.95 (1,678) | 20.73 - 80.81 |  | 30.84 (970) | 14.62 - 61.66 |  | 37.88 (1,193) | 25.22 - 72.01 |  | 48.03 (1,502) | 23.67 - 95.69 |
| Hip replacement | 375.99 (3,561) | 274.62 - 487.78 |  | 378.25 (3,595) | 184.99 - 420.85 |  | 343.37 (3,271) | 259.62 - 443.87 |  | 367.56 (3,511) | 303.08 - 476.09 |
| Hysterectomy | 124.85 (2,069) | 80.56 - 233.04 |  | 101.00 (1,659) | 49.97 - 190.65 |  | 107.82 (1,769) | 63.87 - 170.48 |  | 109.25 (1,777) | 29.10 - 210.27 |
| Inguinal hernia repair | 301.41 (9,552) | 220.08 - 435.66 |  | 185.00 (5,818) | 130.81 - 249.37 |  | 212.64 (6,696) | 132.05 - 356.22 |  | 272.58 (8,525) | 206.02 - 367.17 |
| Knee replacement | 233.79 (7,409) | 168.61 - 341.20 |  | 186.59 (5,868) | 124.43 - 287.57 |  | 209.02 (6,582) | 154.26 - 308.48 |  | 230.76 (7,217) | 166.70 - 305.31 |
| Laparoscopic cholecystectomy | 168.34 (5,335) | 111.99 - 280.28 |  | 104.90 (3,299) | 60.34 - 151.20 |  | 125.34 (3,947) | 93.62 - 220.54 |  | 164.32 (5,139) | 118.70 - 228.09 |
| Tonsillectomy | 163.23 (915) | 45.35 - 301.72 |  | 72.30 (396) | 39.62 - 141.75 |  | 73.19 (398) | 15.56 - 143.62 |  | 98.18 (526) | 58.66 - 204.61 |
| Vein stripping | 61.09 (1,936) | 9.30 - 133.65 |  | 25.47 (801) | 0.00 - 71.21 |  | 35.47 (1,117) | 4.27 - 82.64 |  | 37.03 (1,158) | 18.35 - 111.58 |

**Fig. A1** Boxplots of annual crude utilizations rates (per 100,000 residents) by surgical procedure


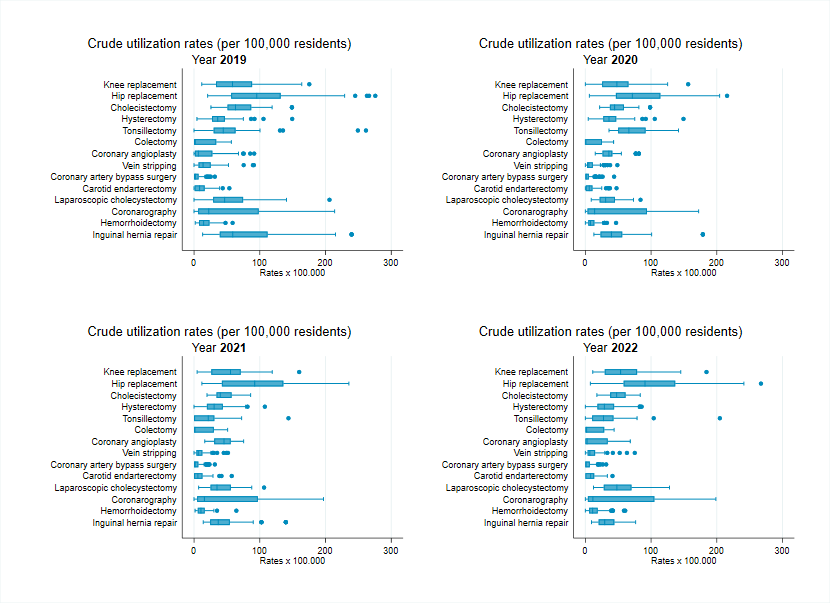


To mitigate the bias associated with measures heavily influenced by extreme values such as the range or interquartile range used in constructing boxplots, we calculated a more robust statistic, the SCV. Again, this analysis proved that geographic variation is ubiquitous and persistent over time (Table A3). For the sake of synthesis, we will exclusively focus on the regional results. The results obtained for the LHAs should be interpreted consistently. In 2022, the procedures that exhibited the greatest SCV at the regional level were CABG (52.34), vein stripping (47.11), hemorrhoidectomy (12.89), tonsillectomy (9.74), carotid endarterectomy (9.53), coronary angiography (6.30), hysterectomy (6.09). According to the classification by McPherson et al. [56], all these SCV values fall within the high or very high category. Analyzing the temporal trend reveals consistently high geographical variation across all four years for these procedures. When comparing 2022 values to pre-pandemic levels, SCVs indicated increased geographical variation for coronary angiography (rising from 5.13 to 6.30, entering the very high variation category) and vein stripping (escalating from 31.96 to 47.11). In contrast, CABG (from 50.74 to 52.34), hemorrhoidectomy (from 11.15 to 12.89), and hysterectomy (from 6.16 to 6.09) remained relatively stable. Notably, reduced variation was found for carotid endarterectomy (declining from 15.16 to 9.53) and tonsillectomy (reducing from 16.09 to 9.74), falling from very high to high variation category. There was a moderate yet improving variation for colectomy (from 5.11 to 4.81, despite a slight increase from 4.57 in 2021), coronary angioplasty (from 5.00 to 3.41), laparoscopic cholecystectomy (from 4.46 to 3.06), and moderate and almost stable variation for knee replacement (from 3.32 to 3.85).

**Table A3** Systematic Component of Variation (SCV) 2019-2022 across 14 elective surgeries

| **Health authority** | **Procedure** | **2019** | **2020** | **2021** | **2022** |
| --- | --- | --- | --- | --- | --- |
| **Tuscany Region** | **Carotid endarterectomy** | **15.16†** | **35.35†** | **11.96†** | **9.53*** |
| LHA1 | Carotid endarterectomy | 17.31† | 70.43† | 6.66* | 8.26* |
| LHA2 | Carotid endarterectomy | 8.32* | 7.35* | 4.27 | 0.14 |
| LHA3 | Carotid endarterectomy | 14.76† | 36.77† | 20.31† | 10.71† |
| **Tuscany Region** | **Cholecystectomy** | **3.92** | **3.35** | **4.29** | **2.36** |
| LHA1 | Cholecystectomy | 1.16 | 2.38 | 3.24 | 1.93 |
| LHA2 | Cholecystectomy | 5.34 | 2.95 | 8.28* | 1.46 |
| LHA3 | Cholecystectomy | 3.18 | 3.62 | 0.29 | 2.80 |
| **Tuscany Region** | **Colectomy** | **5.11** | **5.47*** | **4.57** | **4.81** |
| LHA1 | Colectomy | 6.46* | 6.74* | 6.59* | 13.88† |
| LHA2 | Colectomy | 0.00 | 3.23 | 2.61 | 0.07 |
| LHA3 | Colectomy | 10.68† | 7.32† | 2.03 | 3.28 |
| **Tuscany Region** | **Coronary angiography** | **5.13*** | **6.00*** | **6.77*** | **6.30*** |
| LHA1 | Coronary angiography | 5.14 | 1.15 | 1.67 | 3.33 |
| LHA2 | Coronary angiography | 5.01 | 4.45 | 4.92 | 3.00 |
| LHA3 | Coronary angiography | 2.34 | 3.04 | 4.42 | 6.92* |
| **Tuscany Region** | **Coronary angioplasty** | **5.00** | **10.82†** | **8.39*** | **3.41** |
| LHA1 | Coronary angioplasty | 3.29 | 15.45† | 8.15* | 1.95 |
| LHA2 | Coronary angioplasty | 7.36* | 12.60† | 7.47* | 3.54 |
| LHA3 | Coronary angioplasty | 1.07 | 2.47 | 9.87* | 6.74* |
| **Tuscany Region** | **Coronary artery bypass surgery** | **50.74†** | **112.03†** | **65.84†** | **52.34†** |
| LHA1 | Coronary artery bypass surgery | 11.02† | 52.74† | 15.73† | 10.55† |
| LHA2 | Coronary artery bypass surgery | 96.72† | 31.93† | 85.09† | 21.41† |
| LHA3 | Coronary artery bypass surgery | 49.10† | 25.09† | 23.42† | 54.23† |
| **Tuscany Region** | **Hemorrhoidectomy** | **11.15†** | **56.45†** | **33.68†** | **12.89†** |
| LHA1 | Hemorrhoidectomy | 3.83 | 32.78† | 11.60† | 0.68 |
| LHA2 | Hemorrhoidectomy | 10.08† | 39.53† | 18.16† | 6.61* |
| LHA3 | Hemorrhoidectomy | 6.69* | 31.13† | 25.83† | 14.79† |
| **Tuscany Region** | **Hip replacement** | **1.33** | **2.08** | **0.47** | **0.43** |
| LHA1 | Hip replacement | 0.55 | 0.32 | 0.00 | 0.02 |
| LHA2 | Hip replacement | 0.41 | 2.66 | 1.08 | 0.55 |
| LHA3 | Hip replacement | 1.36 | 2.32 | 0.58 | 0.68 |
| **Tuscany Region** | **Hysterectomy** | **6.16*** | **7.78*** | **4.13** | **6.09*** |
| LHA1 | Hysterectomy | 3.62 | 6.13* | 0.84 | 0.00 |
| LHA2 | Hysterectomy | 0.85 | 4.54 | 0.00 | 0.00 |
| LHA3 | Hysterectomy | 6.02* | 6.53* | 0.00 | 2.51 |
| **Tuscany Region** | **Inguinal hernia repair** | **2.67** | **2.82** | **5.88*** | **2.44** |
| LHA1 | Inguinal hernia repair | 1.97 | 3.55 | 5.57* | 2.14 |
| LHA2 | Inguinal hernia repair | 2.39 | 1.78 | 4.27 | 0.85 |
| LHA3 | Inguinal hernia repair | 2.52 | 1.05 | 1.50 | 2.14 |
| **Tuscany Region** | **Knee replacement** | **3.32** | **3.95** | **2.68** | **3.85** |
| LHA1 | Knee replacement | 1.01 | 2.94 | 3.99 | 2.19 |
| LHA2 | Knee replacement | 2.85 | 1.28 | 1.98 | 2.25 |
| LHA3 | Knee replacement | 3.85 | 6.28* | 2.05 | 4.91 |
| **Tuscany Region** | **Laparoscopic cholecystectomy** | **4.46** | **4.10** | **5.38** | **3.06** |
| LHA1 | Laparoscopic cholecystectomy | 1.20 | 2.56 | 4.04 | 2.25 |
| LHA2 | Laparoscopic cholecystectomy | 5.29 | 2.45 | 9.93 | 2.16 |
| LHA3 | Laparoscopic cholecystectomy | 4.19 | 5.39 | 0.89 | 3.49 |
| **Tuscany Region** | **Tonsillectomy** | **16.09†** | **25.80†** | **6.66*** | **9.74*** |
| LHA1 | Tonsillectomy | 1.96 | 20.88† | 12.72† | 0.00 |
| LHA2 | Tonsillectomy | 9.01* | 0.00 | 2.49 | 0.00 |
| LHA3 | Tonsillectomy | 6.03* | 15.77† | 0.00 | 6.17* |
| **Tuscany Region** | **Vein stripping** | **31.96†** | **129.11†** | **48.55†** | **47.11†** |
| LHA1 | Vein stripping | 118.63† | 196.66† | 93.96† | 17.63† |
| LHA2 | Vein stripping | 16.23† | 79.47† | 24.30† | 27.33† |
| LHA3 | Vein stripping | 7.15* | 18.12† | 18.80† | 20.11† |

† Very high variation (SCV>10)

* High variation (SCV between 5.4 and 10)

**Table A4** Socio-demographic characteristics across Italian regions, 2022

| **Italian region** | **Average age** | **Percentage of male population** | **Percentage of female population** | **Legal marital status (percentage celibate)** | **Legal marital status (percentage nubile)** |
| --- | --- | --- | --- | --- | --- |
| Abruzzo | 46,5 | 48,92% | 51,08% | 46,20% | 37,09% |
| Basilicata | 46,3 | 49,24% | 50,76% | 45,90% | 36,98% |
| Calabria | 45,0 | 48,96% | 51,04% | 46,37% | 37,94% |
| Campania | 43,1 | 48,85% | 51,15% | 47,16% | 39,88% |
| Emilia-Romagna | 46,2 | 48,89% | 51,11% | 48,92% | 40,16% |
| Friuli-Venezia Giulia | 47,6 | 48,78% | 51,22% | 47,26% | 37,14% |
| Lazio | 45,5 | 48,44% | 51,56% | 48,63% | 40,92% |
| Liguria | 48,9 | 48,09% | 51,91% | 45,89% | 35,88% |
| Lombardia | 45,4 | 49,10% | 50,90% | 48,63% | 39,84% |
| Marche | 46,8 | 48,78% | 51,22% | 47,24% | 37,66% |
| Molise | 47,3 | 49,29% | 50,71% | 45,69% | 36,18% |
| Piemonte | 47,1 | 48,72% | 51,28% | 46,64% | 37,06% |
| Puglia | 45,2 | 48,71% | 51,29% | 45,19% | 38,32% |
| Sardegna | 47,6 | 49,05% | 50,95% | 49,41% | 41,00% |
| Sicilia | 44,4 | 48,70% | 51,30% | 45,89% | 38,34% |
| **Toscana** | **47,2** | **48,50%** | **51,50%** | **46,82%** | **38,23%** |
| Trentino-Alto Adige/Südtirol | 43,8 | 49,41% | 50,59% | 51,98% | 43,57% |
| Umbria | 47,3 | 48,35% | 51,65% | 46,01% | 37,09% |
| Valle d'Aosta/Vallée d'Aoste | 46,4 | 48,97% | 51,03% | 50,00% | 40,10% |
| Veneto | 45,9 | 49,11% | 50,89% | 47,72% | 38,88% |
| **Italian average** | **47,3** | **48,82%** | **51,18%** | **47,48%** | **39,05%** |

Data source: Istituto Nazionale di Statistica (Istat) (2022). Accessed on 1^st^ July 2025. Retrieved at: https://esploradati.istat.it/databrowser/
